# Lateral Pressure of the Teeth Considered in Relation to Obscure Nervous Affections

**Published:** 1859-01

**Authors:** J. M'Calla

**Affiliations:** Lancaster, Pa.


### ARTICLE XVI.

*Lateral Pressure of the Teeth considered in relation to obscure Nervous Affections. Read at the Fourth Annual Meeting of the "American Dental Convention," by* J. M'CALLA, D. D. S., *Lancaster, Pa.*

THE evils resulting from undue lateral pressure of the teeth, have not been sufficiently dwelt upon by authors on dental pathology. Crowded and badly arranged dentures have usually been spoken of, as furnishing conditions favorable to decay, while the more obscure affections to which they frequently give rise have either been allowed to pass unnoticed, or have been attributed to other causes. Periostitis, abscess, necrosis of the jaw bones, and tetanus, are some of the more obvious results of this condition of the teeth; while otalgia, epilepsy, hysteria, and neuralgia in some of its varied phases, though equally frequent, are more liable to be regarded as arising from general debility, or a diseased condition of the system.

The nervous disturbance is frequently so distant from the primary cause as to present serious obstacles to a correct diagnosis. The toothache of pregnant women, unaccompanied with caries, is an instance of this kind, while the sympathetic or reflex action of the fifth and seventh pairs upon the uterine nerves, has in some rare cases, produced abortion. The object of the present effort is more for the purpose of directing the attention of the profession into that channel, than from any desire to attempt to fill up the hiatus; we shall therefore present the most available evidence that comes within our reach, from the experience of others, in connection with the result of our own observations during a series of years.

Velpeau relates several cases in which mal-position of the wisdom tooth, was followed by necrosis and exfoliation of large portions of the inferior maxilla, fungous growths, immobility of the jaw, epilepsy and insanity. In one case of extensive abscess, the communication with the surface, was by means of a sinus extending down to the clavicle. Some of these cases had been previously treated as rheumatic affections, others as syphilitic ; in one case the surgeon was about to extirpate the right tonsil, when the true cause of the patient's suffering was accidentally discovered to be an advancing wisdom tooth.

Mr. Tomes upon this subject says: "It is obvious that the evils produced by the development of irregularly placed wisdom teeth, arise wholly from pressure produced by the gradual growth of the dental pulp, preparatory to its calcification ; by which the super-imposed tooth is forced against the tissues by which it is covered. In the natural course of things, these tissues are absorbed, and make way for the rising tooth ; but if they are not absorbed, and, therefore, resist the advance of the tooth, the pressure reacts through the tooth upon the developing pulp. We all know that pressure upon a nerve or nerves, though slight in degree, will produce more or less pain, and that the pain will be felt in the part to which the fibres pressed upon are ultimately distributed. Instead, then, of being surprised that interruption to the growth of a tooth should produce pain, extending through the whole jaw, or to the side of the head, or about the ear, we should be led to infer that such phenomena would exist.

It would be, perhaps, difficult to estimate the actual amount of mechanical force developed by a growing tooth, but we may form some estimate, by observing the force generated by a growing vegetable. We have, most of us, seen a tolerably weighty piece of stone raised by the growth of a plant beneath, any fibre of which might be readily crushed between the thumb and finger.

Mr. C. Spence Bate, in an article on irregular teeth says: "Perhaps of all the evils which result from irregularity in the teeth, none are so painful and serious in their results, as that which is possible to ensue from the abnormal position of the third molar, and which from some cause—probably dependent upon our artificial habits,—is far more frequently irregularly planted in the jaw, among civilized tribes, than otherwise ; and often, when perfectly in a line with the rest, they impinge so tightly against their neighbor, as to be a source of pain, not unfrequently amounting to acute neuralgia. This I believe to be the true source of a mysterious toothache, liable to be confounded with neuralgia, but which the best writers on dental surgery are either silent about, or candidly confess their inability to fathom, owing to the conception of the existence of pain being seated far from the origin.

CASE 1.—June, 1849, a lady suffering from an obscure nervous affection, was introduced by my friend, the late Dr. Geo. B. Kerfoot, of Lancaster. He had gone through the usual list of remedies, without any sensible benefit to the patient, and now passed her over to me with a view to discover whether or not, the trouble might be ascribed to a diseased condition, or faulty arrangement of the teeth. The second and third, right and left inferior molars were found much decayed ; the wisdom teeth, particularly, were broken down to the edge of the gums, and a source of irritation to the surrounding soft structures ; the inflammation consequent upon this state of affairs had extended to the peri-dental membrane, the ramii* of the jaw posteriorly, and the second molars in front impinged so closely upon the wisdom teeth, as to prevent anything like expansion in either of these directions, while the second molar extending above and overhanging the wisdom teeth, prevented upward movement ; hence the display of nervous irritability. I advised the extraction of the four teeth named, and proceeded at once to do so ; the patient however, concluded to have but one side cleared at a time. So much relief was experienced by the operation, that she came back in the course of a few days to say that I judged correctly, and she would now submit to the removal of the other two. I have seen, and op-

* Sometimes erroneously termed, coronoid process.

erated for her repeatedly since then, and can therefore certify that she has had no return of neuralgia.

Case 2.—July, 1849, Mr. J. F. H., aged 24, called my attention to the condition of his mouth, but more especially to the posterior lateral region. He complained of something, not amounting to actual pain, but an uneasiness which constantly served to direct his attention to the left inferior wisdom tooth. Upon examination it was found to be just emerging through the gum, but under rather straightened circumstances, there was not sufficient room for its proper arrangement in the arch, and as the force from behind appeared to be capable of overcoming all obstacles to its onward progress, it was forced to take a position either outside, or inside of the regular arch. In the latter position we found it, nearly covered with a flap of gum, highly inflamed, and the under surface, or that in immediate contact with the tooth in a state of suppuration, in consequence of which the breath was so much contaminated, as to become a source of annoyance to himself, and those with whom he came in contact. I scarified the gum freely, prescribed a suitable wash, and requested him to let me see it occasionally. A few months afterwards he called to say, that the other lower wisdom tooth was in a condition similar to the first, only that the pain was much more severe. I found them both sufficiently advanced to be readily arrested, and therefore advised their immediate removal, which was accordingly done. I had frequent opportunities of seeing the gentleman afterwards, and found that his freedom from pain, and freedom from the teeth, took place at the same time. As the superior wisdom teeth advanced, however, another round of troubles ensued, similar to those we have just described ; these were also removed, and with them, the cause. All four of the teeth were sound at the time of removal, which forces us to the conclusion that the sufferings of the patient resulted from lateral pressure, consequent upon the want of room for the proper eruption and regular arrangement of the wisdom teeth.

CASE 3.—Mr. Charles Bew, in his work on tic-douloureux, London, 1824, presents a number of cases of this disease, clearly attributable to lateral pressure, or undue contact of the teeth. He says "tic-douloureux seldom makes its attack till that season, or period, in which nature has accomplished the usual compliment of teeth for the support and service of the masticating system, and is oftentimes solicited, or aided in its approach, by the insufficiency of space in the jaw destined for their reception. This observation is but too often demonstrated by the derangement, and disease of the *dentes sapientiæ*. Of the many persons I have seen, and relieved from tic-douloureux, all were uniform in the description of the *darting pains,* and uneasy sensations in the vicinity of these teeth, in the first instance, and a sure succession of periodical plunging and twitching of the nerves of the face, nearest in situation to the teeth affected. We give one case in illustration :—A northern baronet of the highest respectability residing near Kelso, for many weeks suffered misery of a distressing description, from pain proceeding from the angle of the upper jaw, and diverging towards the nose and eyes. His medical advisers attributed it to rheumatic affections of the face, and proceeding on this opinion, the pains were endured with the assistance of palliatives, until endurance was no longer endurable, and the required relief was sought for elsewhere; and, although achieved with difficulty, and danger, proved fortunately effective. A travelling dentist had been much talked of in the town, and was desired to attend the baronet at his seat. When there arrived, the finger of the patient was placed on the spot whence the pain was conceived to arise, and on inspection, was pronounced by the dentist to proceed from the dens-sapientiæ, though candor compelled him to declare *he could see no fault in it;* when just at the moment the pain returning with renewed vigor, immediately fixed the baronet's resolution for its removal. Unfortunately, by some mismanagement, or the application of a greater degree of force than was requisite, the expulsion of the pain-exciting tenant was not affected, but at the expense of a great portion of the fixtures adjacent; and though this was attended with some little inconvenience, he had the comfort to find, the long-dreaded *darting* had entirely disappeared, and left him comparatively happy; until the arrival of a similar occurrence on the opposite side. And very properly considering he was about to go through a second edition of his former sufferings, sought after relief from more skillful hands by removal to Edinburgh; and has since appeared to be (as far as regards tic-douloureux) in perfect peace.

Case 4.—Mr. Liston, the celebrated English surgeon, says —"sometimes immense mischief arises from the irregular growth of the teeth, and patients have even lost their lives in consequence of it. My friend, Mr. Nasmyth of Edinburgh, a most accomplished surgeon and dentist, who took the right way of studying his profession, having been for years a demonstrator of anatomy, met with the following case:—An old friend applied to him with an extensive abscess extending down to the clavicle. His mouth could not be opened, the inflammation locked the jaw, and the patient died. On a post-mortem examination it was found that the cause of the whole mischief was a wisdom tooth growing forward, and lying horizontally, instead of perpendicularly. This is a rare case, but it shows that much mischief, and serious consequences may arise from a trifling cause."

Case 5. 1851.—Mr. J. R. presented himself for the purpose of having a left inferior lateral incisor extracted. His sufferings had been gradually augmenting, for some days previous to the time I was first made acquainted with his condition. He informed me that nothing but dire necessity could induce him to occupy a dentist's chair, but now that time had come. Upon examination, I found the tooth designated, perfectly sound, as were all those in the neighborhood. Supposing the tooth to be under the influence of a reflex or sympathetic action, and the cause to be found elsewhere, I extended the examination to all the teeth in both jaws, but was forced to return to the starting point without discovering anything that would throw light upon the subject. The first left inferior molar was absent, and but for this fact, it is probable I would have suspected lateral pressure as the cause, at an earlier period in the examination. The least pressure upon the tooth was painful in the extreme, and the patient became vociferous in his demands to get rid of it. An explanation of what I now believed to be the cause of his pain, induced him to submit to the employment of the file instead of the forceps. A clear space was made with the instrument between the affected tooth and canine, which removed the pressure and effected a speedy and permanent cure.

CASE 6. 1852.—Mr. B. C. applied to me for advice in relation to the cause of pain in his jaws, which he described as generally worrying, though sometimes acute. He supposed that quite a number of his teeth were carious, and some two or three so much so, as to require extraction. Before proceeding to an examination, I told him there must be some mistake, as I had a distinct recollection of having left his teeth in complete order only a few months previous, and knew from their character, that the ravages to which he alluded could only exist in imagination. Upon inspection, the prediction was verified; no nook could be found, which could raise a doubt as to their soundness. The description of the pain, taken in connection with the age of the patient, (about 24 years) led me to suspect the wisdom teeth as having some agency in the trouble. None of them were erupted, but the tumid and inflamed appearance of the gum behind the second right inferior molar, gave evidence of an advancing tooth. I made a free incision into it, and requested him to let me occasionally see how the case progressed. About six months afterwards he paid me another visit, after having passed a sleepless night, in consequence of a return of his former trouble, now very much aggravated in character. No part of the tooth was yet to be seen; there was little or no tension, or inflammation of the gum, head hot, and considerable flow of saliva. It was now evident that the tooth had been developed in the ramus of the jaw, for want of sufficient space in the body of the bone, and the presentation being nearly horizontal, brought the entire expulsive force against the posterior surface of the second molar. I advised local depletion, the hot foot-bath, and whatever would tend to diminish or divert the action from the affected region. By these means the patient was enabled to enjoy a season of repose, which lasted the greater part of a year. During this time the tooth had changed position ; a portion of it was now visible, forming an obtuse angle with, and pressing hard upon the tooth in front. It is unnecessary to describe the minutiæ of the operation of extraction ; let it suffice, that it was accomplished with much difficulty. But the relief which followed, amply compensated for the pain endured during the process.

CASE 7.—Mr. Davis records in the *London Lancet* of Aug., 1846, the case of a lady, who, six days after labor, was attacked with a severe pain in the region of the uterus. It was of an intermittent character, and during the intermissions she had pain in the face. Leeches, fomentations, etc., were applied to the abdomen, and calomel and opium given internally. He remarks : "The pain in the face now became more severe, and the patient, supposing it to arise from toothache, surrounded the parts with flannel. This circumstance led to inquiries on my part, which ended in an examination of the mouth, when I found the posterior part of the gum, red and swollen. The mystery was explained ; one of the dentes sapientiæ, the cause of all the pain in the uterus, was coming up. The gum was freely lanced ; and the pain in the uterus from that moment, subsided. No more medicine was required or given."

Dr. W. Tyler Smith of London, says : "Irritation of the *trifacial nerve* seems, in rare cases, to excite abortion. It happens when no cause can be recognized but the appearance of the dens sapientiæ, and this phase in dentition is known to produce considerable local, and constitutional disturbance. General convulsions may, in fact, be excited from this source, either in the male or female subject. The reflection of irritation from the trifacial upon the uterine nerves, in young pregnant women, is no more remarkable than the strangury excited by teething, in the infant."

The lamented Prof. Handy, in considering the relation between the dental organs and uterus, says : "when the question is asked, how is it, that an organ sympathizes with some one, in preference to another? we look for the answer, and reply, that it is either owing to its contiguity, or is concerned in the performance of the same function, and hence indicating the closest fellowship and most intimate relation. But in the case of the teeth and uterus there is no such relation, either by contiguity, by direct intercourse, through nerves, or by any special connection in the performance of function. Yet it is manifest there must be a relationship, through some channel or other, to explain the presence of symptoms, which clearly flow as cause and effect, and as clearly point to the dependency which evidently exists between these two sets of organs. Then the question is, what is this channel of intercourse? A man has locked jaw, from injury of the toe, a sympathy even more remote, and quite as obscure as that of the teeth and the uterus ; and no one questions this relationship—but how does it exist? Now, our own opinion is, that the medium of connection is the same in both cases, and that the sentient tract of the spinal marrow is the medium. In the one case, the sentient nerves of the uterus, being impressed by the changes occurring in this organ, at the period of menstruation and pregnancy, reflect this impression to the sentient tract of the spinal marrow, whence it is conducted upwards, along this tract, to the point where the fifth pair of nerves pass off; and here it takes the route of this nerve—the great sentient nerve of the teeth and face—going along the dental branches where there is pain in the teeth, and, by a reflex action, to the muscles of the lower jaw, in the case of *trismur*, where the impression from the sentient nerves of the toe are also reflected to the spinal marrow.

CASE 8.—July, 1854, Mrs. H. L. Z. called for advice in regard to some dental trouble, which she was unable to account for. She described the pain as being unlike toothache, yet so severe as to deprive her of rest at night. She had employed opiates by the advice of her physician, but the relief thus obtained was temporary, and of short duration. The second left inferior molar was thought to have some agency in the trouble. Upon inspection I found the lower arch complete, with the exception of the wisdom teeth, and no appearance of their approach could be discovered. The teeth all sound and regular, though much crowded. I attributed her sufferings to lateral pressure of the teeth, but whether occasioned by an advancing wisdom tooth, or periostitis resulting from some other cause, it was not so easy to determine. The passage of a file between the first and second molars, would have removed the point of resistance, but as less harm would result from an exploration behind the second molar with a sharp lancet, this was the course adopted, and revealed the presence of the wisdom tooth, deeply imbedded in the jaw. A gentle aperient was ordered, in addition to the hot pediluvium, before retiring at night, and this, in connection with the local depletion, served to give a respite of several weeks. The pain now assumed more of the neuralgic character, distributing its agonizing pangs along the different branches of the facial nerve, from the battery of the *pes aserinus*, and producing a degree of prostration, from which she only recovered to meet another attack of the same fearful character. The removal of the second molar seemed to present the best prospect of relief; but this she refused to submit to, indulging the hope, that by the employment of milder means for a time, the wisdom tooth would become sufficiently advanced to be easily extracted, and thus preserve the most important tooth of the two. A few more paroxysms, however, brought her back for relief upon any terms. I extracted the second molar, but found an unsuspected impediment to its removal; the posterior root diverging very much from a straight line, passed under the crown of the wisdom tooth, while it impinged closely upon the angle thus formed in the second molar. Here was a lock without a key. There was some motion towards the cheek and tongue, but no extraction; indeed, the extreme nervous susceptibility of the patient forbade the employment of harsh measures. The movement given to the tooth, it was hoped, might be sufficient to enable the wisdom tooth to glide past the point of resistance, and with this object in view, it was abandoned for the present. For several weeks there was no return of the trouble. Upon her next visit, I found the wisdom tooth making its appearance through the gum, which I lanced thoroughly; and during the six months following, this operation was frequently repeated by her physician and myself, and always afforded relief. But to get entirely rid of the trouble, was now her most anxious desire. The change in position which had taken place since the former attempt at extraction, was so trifling, as to give but little hope of success now, yet the attempt must be made. With a stout bladed lancet, a portion of the inner wall of the alveolus was removed from the second molar, the tooth arrested and luxated by turning it towards the cheek, sufficiently to enable the points of root to mount over the part from which the alveolus had previously been removed, and raised sideways from between the other two. The operation was followed by an entire cessation of the neuralgic affection. Four years have elapsed since then, but no return of the trouble.

The importance of a thorough knowledge of the various evils resulting from the condition of the teeth now under consideration, is strikingly illustrated by a case to be found in Prof. Harris' Principles and Practice. The subject, Mr. D. M., after years of suffering, consequent upon efforts at the eruption of a wisdom tooth, sought relief by a change of climate, in addition to the best medical advice. Both, however, proved unavailing; the irritation extended to the lungs, and established a confirmed phthisis pulmonalis, from which he was only relieved by death.

A record of three cases of abscess of the gums and cheek, two of which opened externally, first appeared in the London *Lancet* of March, 1857, and subsequently in the *Dental News Letter,* Philadelphia, January, 1858. In one of the cases, various remedies had been tried in vain, by different physicians, until Prof. Syme of Edinburgh, discovered that the abscess arose from a wisdom tooth, coming up in the jaw, for which there was not sufficient room. The extraction of the second molar was followed by the gradual subsidence of the swelling ; the wisdom tooth soon filled up the vacant space, and the patient was permanently cured.

Case 9. 1856.—A German aged about forty-five, suffering from what he supposed to be toothache, called upon me for the purpose of having one or two teeth extracted. Upon inspection it was found that the entire denture was present, and every tooth sound, but so jammed together and abraded as to present the appearance of one continuous block. The first and second left superior molars were pointed out as the seat of the pain ; periostitis produced by lateral pressure was clearly indicated ; separation with a file was the only treatment necessary. About one year afterwards he called to have a similar operation performed on the opposite side of the jaw, which speedily relieved him of pain, as on the former occasion.

Case 10. 1858.—A gentleman aged about fifty, called upon me early one morning in February, for advice in regard to the cause of his having lost a night's rest. He walked up and down the floor as one demented, holding on firmly to the aching jaw. Upon examination, I found the upper arch unbroken, with the exception of the first right superior molar, which had been removed about one year previous to this time. The finger of the patient was placed upon the second right superior bicuspid, as the offender. There being no appearance of decay present, I made pressure upon the tooth in the direction of the space left vacant by the absent molar. This aggravated the pain, and at once suggested the remedy. The passage of a file between the first and second bicuspid, was followed by instantaneous relief.

The two following cases are condensed from Desirabode's work on the teeth, translated from the French, for the American Library of Dental Science.

Case 11.—In 1822, Dr. Olmade was attacked with odontalgia, with which he was tortured for three months. The violence of the symptoms was such that his mouth was closed spasmodically, and he was compelled to subsist entirely upon liquid aliment. Having had occasion to see him, at the time his sufferings commenced, it was observed that one of the wisdom teeth of the lower jaw was on the point of coming through. To this cause alone the disorder was attributed, and the patient was advised to have it extracted. Dr. Olmade deferred submitting to the operation until an abscess had formed, which caused a fistulous opening through the cheek. At the same time a portion of the maxillary bone became necrosed, and some fragments escaped with the pus. Becoming convinced that he could obtain no relief from medicine, he determined to follow our advice. The condition of the parts contiguous, was now such as to add very much to the difficulty of the operation. It was therefore found necessary first, to remove the second molar, the crown of which was almost destroyed. The roots of this tooth were anchylosed to the alveolar walls in their whole extent, although they were very short. It was this anatomical arrangement to which was to be attributed all the sufferings of the patient. The wisdom tooth, in fact not having room enough between the angle of the jaw and the second molar, and not being enabled to displace the latter, it had taken an almost horizontal direction from behind forward, so that the posterior face of the crown presented upward. A short time after we effected the extraction, the abscess and fistula closed, but the cure was not completed till after the lapse of two months.

Case 12.—The subject of this case was the son of a rich merchant of Bordeaux, who had been obliged to submit to the extraction of the first right inferior molar, which was deeply excavated by decay. The dentist who was entrusted with the operation, broke the tooth off close to the gum, and consequently left all the root in the alveolus. Two years passed away, during which the patient experienced no inconvenience from the root; but at this time, (he was then twenty-two years of age) he felt a constant sensation of tension, not only immediately about the root, but in the adjacent parts, particularly towards the back part of the mouth. It was not long before this feeling of tension took the character of pain, and this pain in a short time became very troublesome. The dentist who had broken the tooth was again consulted, and not doubting that the presence of the root was the cause of the pain, he attempted its extraction, but all his efforts were fruitless. He then advised the extraction of the adjoining tooth, which closing up the orifice of its alveolus, prevented the extraction of the root. The father of the young man, not believing that it was necessary to extract a sound tooth, in order to effect the extraction of a root, and fearing for this one the fate of the former, refused to permit the operation, and advised his son to wait till their arrival in Paris, to which city they were called by business, so that they might consult in the care of some of our celebrated surgeons. On their arrival in Paris, they consulted Dupuytren. This illustrious practitioner had scarcely examined the mouth of the young man, when he perceived, from the deviation of the tooth, which covered the root, that the wisdom tooth, obstructed in its eruption, had pushed all in front of it, to such an extent that this latter had been buried in the alveolar tissue, thus causing the incessant pain experienced by the patient. He advised the extraction of the displaced tooth, in accordance with the advice of the Bordeaux dentist, and M. Desirabode, with much difficulty succeeded in performing the operation, which sufficed to bring about a speedy cure, as complete as it was prompt.

Jourdain relates a case in which the subject had suffered five years from hemicrania, accompanied with pain and numbness in the lower jaw. After submitting to depletion, to an unwarranted extent, she was fortunate enough to fall into the hands of M. Petit, who discovered that the trouble arose from a crowded condition of the teeth, there being eighteen instead of sixteen in the lower jaw. By extracting the second right and left inferior molars, he gave complete and permanent relief in twenty-four hours.

Dr. C. T. Cushman, in *Dental News Letter*, vol. 5., presents four well marked cases of periostitis, produced by a crowded condition of the teeth, and concludes, that necrosis is frequently occasioned by the pressure of irregular teeth upon each other, in a maxilla too contracted to admit them in a proper position.

Dr. J. D. White of the *Dental News Letter*, reports a remarkable case of periosteal irritation, in vol. 7, of that journal, which after successfully resisting the treatment of several medical gentlemen, was pronounced a case of "irritability of the periosteal membranes, consequent upon the development of the wisdom teeth," and subsequent treatment proved the correctness of the diagnosis.

Dr. W. H. Baker, in vol. 7, *Dental News Letter*, gives two cases from his note book, illustrating the utility of the file, in the relief of pain, produced by the lateral pressure of teeth, in their development.

Mr. Fox says, "It frequently happens that when either of the *dentes sapientiœ* of the lower jaw occasions pain, that the patient does not suffer so much in the tooth itself, as in the ear. The pain often follows the course of the nerves, being diffused all over the cheek, shooting up to the temple, and affecting the head generally."

In corroboration of this, I will here present a case which recently came to my knowledge.

CASE 13.—June, 1858, Miss L. A. R. applied to me for advice in regard to a neuralgic affection, from which she had been suffering for several months. Her trouble began with pain in the jaw, at first slight in degree, but which gradually became more acute and fugitive in its character; sometimes concentrating its forces in and around the ear, and again shooting across the temples and forehead, and occasionally settling in a decayed tooth. Some months before I first saw her, she called upon her dentist, who removed the first and second left superior molars, which were so much decayed as to induce the belief that they were the cause of her sufferings. This was followed by a complete cessation of the pain, which continued about three weeks. The occurrence of an unusually damp and rainy season, appeared to furnish conditions favorable to its return, and her sufferings for some weeks had been most intense. My attention for some months previous, having been specially directed to the subject of the present paper, it was natural that my eye should first fall upon the region of the left inferior wisdom tooth. I found it striving hard for its proper position in the jaw; the second molar was deeply excavated by decay, and the force from behind, exerted upon the advancing wisdom tooth had lodged its crown in the carious cavity adjacent. I told the patient her trouble was caused by want of space for the wisdom tooth, and advised the extraction of the second molar, it being the least valuable of the two in this instance. To this she readily consented, and I have been gratified to learn within the last few days, that she has been entirely free from neuralgic trouble, ever since that time.

CASE 14.—Dr. Koecker relates the following case:—Miss G., of Philadelphia, about sixteen years of age, was troubled with a complaint in her ears, attended with severe pain, and discharge of yellowish matter from them, difficulty of hearing, much general debility, and great depression of spirits. No means had been omitted to obtain the best medical and surgical advice, but it had been utterly unavailing. Her father, and the greater part of her brothers and sisters, were also hard of hearing, and some of them were sometimes troubled with a like discharge from the ear, though not to such an extent, nor accompanied with such severe pain. The cause was supposed to be some natural defect in the organization of the parts, and the affection consequently regarded as incurable. The teeth were, generally, under the influence of caries, and the disease had penetrated to the cavity in some of them. They were all more or less encrusted with tartar, her gums, and the sockets of her teeth were much inflamed, and bled at the slightest touch. The second left inferior molar was carious and painful, and the opposite one was also carious. The crowns of the second right and left superior bicuspids were nearly destroyed. There was but little room for the dentes sapientiæ, and knowing that in the warm and changeable climate of America, their formation is frequently hastened at a premature age, particularly in delicate and irritable constitutions, I readily suspected that their precocious formation, and the want of room for them to pass through the gums, in combination with the diseases of the teeth, were the causes of the inflammation, and the pain in the ears.'' Dr. K. at once instituted the treatment indicated in the case, and in conclusion remarks: ''These measures were followed by great improvement of the general health, cessation of pain in the ears, as well as the discharge; the hearing became good, but not quite so acute in the left as in the right ear. Before I left Philadelphia, in 1822, I saw the patient very frequently; her health and spirits were good, and her hearing, if not perfect, was as good as that of any other member of her family.''

From the various testimonies which have now been offered, it is evident that numerous nervous disorders are dependent upon the irritation caused by lateral pressure of the teeth, and that these disorders occur most frequently about the time of eruption of the wisdom teeth, in consequence of imperfect development of the maxillary bones, or the too early removal of the deciduous teeth, whereby contraction of the dental arches is produced, and a consequent insufficiency of space for the proper arrangement of the permanent teeth.

In relation to the treatment, it will be unnecessary to extend our remarks, farther than to say, that the various phenomena attending these affections require the closest scrutiny.

Cases frequently occur, so obscure as to evade for a long time, the most searching investigation. A keen discernment and sound judgment are necessary, therefore, on the part of the dentist, to enable him to discriminate between reflex or sympathetic action, and immediate or exciting causes. In this manner alone will he be enabled to direct his remedies to the source, and not the symptoms of the trouble, and vary their character to suit the circumstances of each case ; allaying or subduing inflammatory action, soothing and quieting the nervous sensibilities, and seizing the most favorable opportunity to remove the exciting cause.

[*Dent. Register.*

